# Atypical Social Attention and Emotional Face Processing in Autism Spectrum Disorder: Insights From Face Scanning and Pupillometry

**DOI:** 10.3389/fnint.2019.00076

**Published:** 2020-02-12

**Authors:** Debra L. Reisinger, Rebecca C. Shaffer, Paul S. Horn, Michael P. Hong, Ernest V. Pedapati, Kelli C. Dominick, Craig A. Erickson

**Affiliations:** ^1^Division of Developmental and Behavioral Pediatrics, Cincinnati Children’s Hospital Medical Center, Cincinnati, OH, United States; ^2^Department of Pediatrics, University of Cincinnati College of Medicine, Cincinnati, OH, United States; ^3^Division of Neurology, Cincinnati Children’s Hospital Medical Center, Cincinnati, OH, United States; ^4^Division of Child and Adolescent Psychiatry, Cincinnati Children’s Hospital Medical Center, Cincinnati, OH, United States; ^5^Department of Psychiatry and Behavioral Neuroscience, University of Cincinnati College of Medicine, Cincinnati, OH, United States

**Keywords:** eye tracking, autism spectrum disorder, social attention, emotional faces, pupillometry

## Abstract

Social attention deficits are a hallmark characteristic within autism spectrum disorder (ASD) and have been hypothesized to have cascading effects on emotion recognition. Eye-tracking methodology has emerged as a potentially reliable, feasible, and sensitive biomarker for examining core phenotypic features of ASD; however, these findings are mixed with regards to measuring treatment change in clinical trials. The present study aimed to assess the utility of an eye-tracking paradigm to discriminate between clinical groups in social attention and emotion recognition through face scanning and pupillometry. The present study also assessed the reliability of this paradigm within the ASD sample to further our understanding of the utility of eye-tracking for future clinical trials. Participants included 42 individuals with ASD, 29 developmental disability (DD) controls, and 62 typically developing (TD) controls between 3 and 25 years of age. An emotional faces eye-tracking paradigm was administered to all participants, with the ASD group completing the paradigm a second time approximately 2 months later. Participants’ average proportion of looking and number of fixations to specific areas of interest (AOI) were examined along with changes in pupil reactivity while viewing different emotional faces. Results suggest atypical face-scanning through a reduced proportion of looking and the number of fixations toward the eyes in the ASD group regardless of the emotion that was presented. Further, pupillometry measures were able to detect increases in pupil dilation to happy faces in the ASD group. Lastly, test-retest reliability coefficients varied between the poor and excellent range based on the mechanism assessed, with the proportion of looking demonstrating the highest reliability coefficients. These findings build on the promise of eye-tracking as a feasible and reliable biomarker for identifying social attention and emotion recognition deficits in ASD. Detecting differences in emotion recognition explicitly through facial scanning was not as clear. Specific mechanisms within the eye-tracking paradigm may be viable options for assessing treatment-specific outcomes.

## Introduction

Autism spectrum disorder (ASD) is a neurodevelopmental disorder characterized by significant impairments in social communication, restricted interests, and the presence of repetitive and stereotyped behaviors (American Psychiatric Association, 2013). Within the research on social communication deficits in ASD, there has been specific interest in attention to faces or social stimuli across the lifespan (for review, see Guillon et al., 2014; Chita-Tegmark, 2016). Specifically, it has been hypothesized that deficits in social attention (e.g., reduced attention to social stimuli as a whole or atypical allocation of attention to social stimuli) may cause reduced social processing and a loss of relevant information necessary for the development of appropriate social functioning. Further, these deficits in social attention may also cause difficulty in the interpretation of emotional information (Pelphrey et al., 2002; Wagner et al., 2013). In light of the knowledge surrounding these deficits, there is great interest in identifying and developing feasible, valid, and reliable outcome measures to be utilized in clinical trials that are sensitive to assess the core phenotypic features of ASD. The present study examines the utility of an emotional face eye-tracking paradigm to discriminate between clinical groups in addition to evaluating the reliability of the paradigm in ASD.

To date, there have been many studies conducted examining deficits in social attention through abnormal face scanning in ASD; however, the literature is quite mixed with regards to hypothesized causes and whether these deficits are consistently present. One proposed theory suggests individuals with ASD find attention to eyes over stimulating with a heightened sensitivity to social stimuli to support an eye “aversion” hypothesis (Dalton et al., 2005; Spezio et al., 2007b). Another possibility suggests individuals with ASD experience a reduced reward value for social stimuli (Dawson et al., 2005; Chevallier et al., 2012). Specifically, the social motivation theory implies individuals with ASD may not seek out social stimuli because eye contact and faces are not intrinsically rewarding and may not be activating their cognitive reward systems appropriately. This reduced reward is hypothesized to be causing the failure to attend to faces or to develop expertise to attend to faces, resulting in abnormal attention to faces.

Regardless of the cause, a number of studies have suggested that individuals with ASD spend less time attending to the eyes of faces and more time looking at mouths, bodies, and objects in comparison to typically developing (TD) controls across the lifespan (Klin et al., 2002; Pelphrey et al., 2002; Corden et al., 2008; Riby and Hancock, 2008; Rice et al., 2012; Hanley et al., 2013; Auyeung et al., 2015). In comparison to typical development, attention to faces and social stimuli is expected to emerge during infancy and extend into adulthood, with a preferential bias toward the eyes of faces across a variety of tasks and settings (Birmingham et al., 2008). Unfortunately, a number of studies have found no significant differences in face scanning to particular facial regions between ASD and TD controls (Wagner et al., 2013; Gillespie-Smith et al., 2014; Åsberg Johnels et al., 2014; Kwon et al., 2019). These mixed findings within the ASD literature suggest a lack of consensus on social attention deficits in ASD as measured through eye-tracking paradigms; which could be accounted for by the unknown origin of these deficits, the variability of paradigms utilized, how well these paradigms measure social attention, and how sensitive they are to the core phenotypic features of ASD.

Appropriate social attention through facial scanning is also critical for accurate emotion recognition. Within the TD literature, research has demonstrated different attention patterns in relation to positive vs. negative emotions. For example, TD individuals will fixate more on the eye region of negative emotions in contrast to the mouth region of positive emotions (Eisenbarth and Alpers, 2011; Messinger et al., 2012). In addition to the identified deficits in face scanning patterns in ASD, these deficits are further complicated when adding in emotions. Specifically, deficits in the face-scanning of different simple emotions (e.g., happy, sad, fear) have been identified through abnormal-looking time and a number of fixations toward certain regions of emotional faces (Pelphrey et al., 2002; de Wit et al., 2008). Spezio et al. (2007a,b) hypothesized adults with high-functioning ASD fail to make use of the information from the eyes when interpreting facial expressions; therefore, reduced attention to the eye region of faces may have downstream effects on emotion processing in ASD. Unfortunately, the literature is mixed in supporting this theory. According to Sawyer et al. (2012), they suggest emotion recognition cannot be fully explained by impairments in facial scanning after their results demonstrated impairments during an emotional recognition task in comparison to no impairments with facial scanning of basic and complex emotions in ASD.

Aside from examining facial scanning to assess social attention and emotion recognition impairments in ASD, emotional arousal as captured through the autonomic nervous system (ANS) can also be considered. Pupil reactivity, as measured using eye-tracking pupillometry, has been identified as a reliable indicator of emotional arousal that reflects changes in the brain activity that underlie the cognitive events of emotion processing (Bradshaw, 1967; Bradley et al., 2008; Kret, 2015). Specifically, increased sympathetic activity and decreased parasympathetic activity prompt pupil dilation resulting in pupil diameter increases being mediated by both divisions of the ANS (Steinhauer et al., 2004). More recently, pupil reactivity has been used to assess the ANS in response to social stimuli and emotion recognition in ASD during screen viewing (Falck-Ytter, 2008; Sepeta et al., 2012; Nuske et al., 2014a,b). Similar to the social attention and emotion recognition literature, there are mixed findings with respect pupil reactivity in ASD. Specifically, findings have demonstrated pupil constriction while viewing other children’s faces (Anderson et al., 2006), reduced pupillary responses to fearful expressions of unfamiliar people (Nuske et al., 2014a), and increased pupil dilation while viewing inverted, but not upright, emotional faces (Falck-Ytter, 2008) in young children with ASD. Conversely, some studies have demonstrated no change in pupillary responses when viewing emotional faces (Sepeta et al., 2012; Wagner et al., 2013). Combining pupillometry as a measure of emotional arousal and face scanning as a measure of social attention to emotional faces may provide a clearer picture of emotion recognition processing in ASD; however, very few studies have explored these combined mechanisms.

Social attention has notably been identified as one of the earliest hallmark impairments in ASD with the promise of being a predictive diagnostic biomarker for ASD outcomes (Jones and Klin, 2013; Elsabbagh et al., 2014; Jones et al., 2016). These findings have now pushed the field to begin assessing and identifying effective behavioral and pharmacological treatments that can improve social functioning in ASD. Unfortunately, a significant challenge currently being faced within the ASD treatment literature is identifying reliable, valid, and feasible outcome measures that are sensitive to change in measuring the core phenotypic symptoms in ASD. Until recently, most outcome measures used in ASD treatment research have relied on caregiver report or clinician-administered assessments (Bolte and Diehl, 2013). An explicit interest in the utility of biomarkers to measure treatment change in clinical trials has emerged. A promising start for eye tracking was identified by Murias et al. (2018), where they found a strong association between a social attention eye-tracking task and caregiver reports of social communication frequently utilized in ASD clinical trials. Nevertheless, the theme of variability continues with some findings suggesting eye tracking is sensitive enough to detect treatment effects (Auyeung et al., 2015; Fletcher-Watson and Hampton, 2018) and other findings identifying change through clinical measures with no treatment change detected through eye-tracking (Bradshaw et al., 2019).

The present study aims to expand the understanding of the current literature of eye-tracking as a reliable and feasible biomarker for assessing social attention and emotion recognition using a chronologically diverse ASD sample with mentally and chronologically age-matched comparison groups. The methodology utilized in the present study mimics previous work completed by Farzin et al. (2009, 2011) that demonstrated the feasibility and reliability of an emotional faces paradigm in fragile × syndrome (FXS). Given majority of their sample had a co-occurring diagnosis of ASD (Farzin et al., 2009), this paradigm may show promise within ASD as well. It is hypothesized that individuals with ASD will demonstrate reduced attention to the eye region of different emotional faces that varies across emotions in comparison to the mentally and chronologically age-matched control groups. Further, it is hypothesized that individuals with ASD will exhibit abnormal pupil reactivity to the different emotions presented (e.g., reduced reactivity to fearful faces compared to increased reactivity to happy faces). Last, we anticipate that the paradigm will exhibit good-to-excellent reliability estimates within the ASD sample.

## Materials and Methods

### Participants

Participants were drawn from a larger study examining potential biomarkers in ASD at Cincinnati Children’s Hospital Medical Center. The present study included 42 individuals with ASD (83.33% male), 29 age-, gender-, and IQ-matched developmental disability (DD) controls (89.65% male), and 62 age-, gender-matched TD controls (88.79% male) between 3 and 25 years of age (*M* = 12.33, *SD* = 5.80). Of the sample, 72% were White, 12% were Black, 10% were Other/Multiracial, 3% were Hispanic/Latino, 2% were Asian, and 1% were Native Hawaiian or Other Pacific Islander. The ASD group had a confirmed diagnosis of ASD through a structured clinical interview using the Diagnostic and Statistical Manual of Mental Disorders, Fifth Edition (DSM-5) ASD criteria (American Psychiatric Association, 2013), testing with the Autism Diagnostic Observation Schedule, Second Edition (ADOS-2; Lord et al., 2012), and administration of the Social Communication Questionnaire (SCQ; Rutter et al., 2003). Further, the ASD participants did not have any known syndromic or other genetic variant associated with their ASD diagnosis. The TD control participants had no reported or suspected developmental concerns, fell in the normal range (e.g., between 90 and 125) of cognitive functioning on IQ measures administered through the study, and an SCQ total score less than 15. The DD control group was matched with a subgroup of the ASD participants with an IQ less than 90. The DD control group was also administered the ADOS-2 to ensure none of the participants had undiagnosed ASD. All participants or their guardians provided written informed consent and participant assent (if feasible) for study participation, and the study was approved by the local Institutional Review Board.

Participants’ cognitive functioning was measured across all three groups utilizing the Stanford-Binet Intelligence Scales, Fifth Edition (SB-5; Roid, 2003) or the Differential Ability Scales-II (DAS-II; Elliott, 2007) to obtain a Full-Scale IQ score. One DD control participant and eight ASD participants were not able to complete one of the above cognitive measures due to behavioral concerns or functioning level. This resulted in statistically significant differences between the mean Full-Scale IQ score of the ASD group and the DD control group (*F*_(2,121)_ = 54.70, *p* = 0.000); however, adding in the eight lower functioning individuals would assumedly account for these differences and decrease the ASD mean Full-Scale IQ score. These participants were still included in the original sample due to their ability to complete the eye-tracking task despite their low cognitive abilities. Participants’ caregivers or guardians across all groups completed the SCQ. Participants’ caregivers of the ASD group completed the Aberrant Behavior Checklist (ABC; Aman et al., 1985) and the Social Responsiveness Scale (SRS; Constantino and Gruber, 2005). No significant group differences were found based on chronological age (*F*_(2,120)_ = 2.29, *p* = 0.106). As expected, significant group differences emerged across groups on the SCQ consistent with the lack of ASD diagnosis in the DD and TD control groups (*F*_(2,130)_ = 90.79, *p* = 0.000). See Table 1 for participant descriptive statistics along with the caregiver rating scales for the analyzed sample.

**Table 1:** Descriptive statistics of ASD, DD, and TD variables.

	ASD					DD					TD					Group differences
Variable	*n*	Mean	*SD*	Min	Max	*n*	Mean	*SD*	Min	Max	*n*	Mean	*SD*	Min	Max	*p*
Age (years)	36	14.05	5.80	3.11	21.10	28	11.25	5.17	3.73	24.60	59	11.88	5.96	3.04	25.47	0.106
Full scale IQ	30	88.07	19.71	40.00	137.00	27	73.56	10.09	51.00	88.00	59	104.41	8.99	91.00	124.00	0.000^a^
SCQ	36	19.75	5.72	7.00	33.00	28	10.25	6.17	0.00	24.00	59	5.17	4.85	0.00		0.000^b^
SRS Total T-Score	24	85.54	14.25	57.00	113.00											
ABC Irritability	24	12.42	11.00	0.00	41.00											
ABC Lethargy	24	15.92	7.96	0.00	35.00											
ABC Stereotypy	24	4.96	4.22	0.00	15.00											
ABC Hyperactivity	24	17.96	12.92	0.00	40.00											
ABC Inappropriate Speech	24	5.00	4.12	0.00	12.00											

*Note. Table includes data from the analyzed sample of participants; ASD, autism spectrum disorder; DD, developmental delay controls; TD, typically developing controls; SD, standard deviation; ABC, Aberrant Behavior Checklist; SRS, Social Responsiveness Scale. ^a^Group differences found on full scale IQ (TD > ASD>DD). ^b^Group differences found on SCQ (ASD > DD > TD)*.

### Apparatus and Stimuli

**Figure 1:**
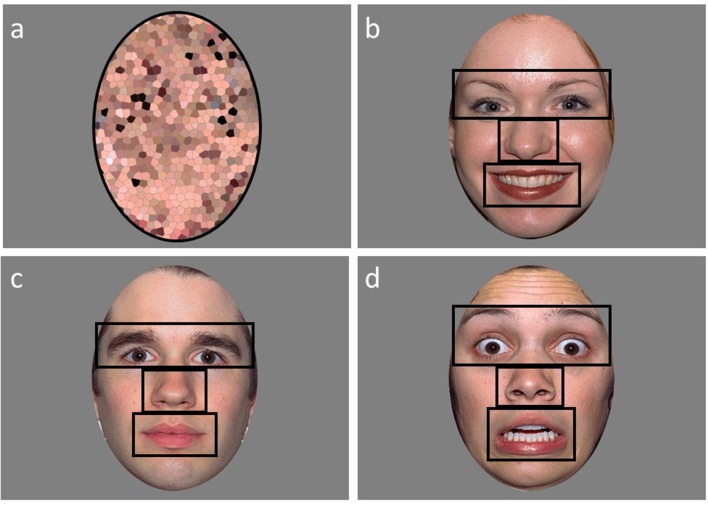
An example of a scrambled **(A)**, happy **(B)**, calm **(C)**, and fearful **(D)** face used in the emotional faces paradigm with the areas of interests (AOI’s) outlined in black.

Eye-tracking data were collected using a Tobii (Stockholm, Sweden) T120 infrared binocular eye tracker sampling at a rate of 120 Hz to record X and Y coordinates of eye position and pupil diameter along with gaze duration. The paradigm was run on an integrated 17-inch flat-panel monitor (1,280 × 1,024 pixels resolution) running Tobii Studio (Version 3.0, Tobii Technology, Sweden). Stimuli consisted of 12 colored photographs of adult human faces (equal numbers of males and females) from the NimStim Face Stimulus Set (Tottenham et al., 2002), each showing a calm, happy, or fearful facial expression (see Figure 1). Each emotional face was presented on the screen for 5 s. Prior to presenting the emotional faces, a scrambled version of the face image was presented for 1 s (Figure 1A). Similar to Farzin et al. (2009, 2011), each face and corresponding scrambled image were matched on mean luminance, and equivalence was confirmed using a photometer (Minolta, LS-100, Osaka, Japan). Face images subtended a 12.12° by 17.19° region (the size of an actual human face) when viewed from a distance of 60 cm, and were presented on a standard 50% gray background (RGB: 128, 128, 128).

### Procedure

All participants were assessed as part of the larger battery during a 1 day visit. Following clinical assessments, participants were allowed a break, if needed or requested, to ensure they were at baseline levels prior to completing the paradigm. Once participants were at their normal baseline state, they were seated in a quiet room in front of the eye tracker at a distance of 60–65 cm from the eye tracker monitor. Each participant was presented with verbal instructions to “look at the screen” or a “first-then” communication tool to demonstrate that the child would first look at the screen and then receive a trivial prize. The eye tracker was calibrated for each participant at the beginning of each session using the Tobii Studio “five-point infant calibration.” Successful calibration was ascertained *via* Tobii Studio’s automated validation procedure. A second attempt to calibrate was conducted if the participant did not successfully calibrate. The task was discontinued if they were not successfully calibrated after two attempts. Following calibration, participants were again instructed to look at the upcoming pictures presented on the screen through verbal instruction or a “first-then” visual prior to the start of the task. Subjects completed one of two variations with different randomizations of the order of emotional faces. Approximately 8–12 weeks later, 19 of the participants in the ASD group returned to the lab to repeat the same battery of measures they received during the first visit. The first and second sessions were the same with respect to the order of the protocol, room set-up, and timing. Depending on which randomization order of the faces the ASD group received at their first visit, they received the other randomized order at the second visit. The second visit in the ASD group allowed us to examine the test-retest reliability of the eye-tracking measure administered. The average length of time between the first and second visits was 9.77 weeks.

### Statistical Analyses

#### Data Extraction

Areas of interest (AOI) for the eyes (including eyebrows), nose, mouth, and other (the rest of the face minus the eyes, nose, and mouth regions) were created (Figure 1). A single ellipse AOI around the face was utilized for the scrambled faces. Two variables were extracted for the analyses from Tobii Studio: fixation count and proportion of looking time to each AOI region. Fixation counts (defined as any data point within a 35-pixel radius for a minimum duration of 100 ms) were calculated by averaging the number of fixations to the AOI regions. The proportion of looking time was calculated by dividing the looking time to the AOI region by the total looking time to face. Not assessed by Farzin et al. (2009, 2011), a proportion of valid looking variable was calculated to assess overall attention during the task in order to exclude participants who had minimal viewing time across the task. The proportion of valid looking was calculated by dividing the total looking time to anywhere on the screen for all the faces divided by the total stimulus presentation time across all faces. Participants were excluded if they had less than 35% valid looking data across the faces. This resulted in six ASD, one DD, and three TD participants to be excluded from the analyses. Two of the six ASD participants that were excluded for valid looking data were also included in the eight ASD participants that could not complete IQ testing. The final sample of participants for the analyses included: 36 individuals with ASD, 28 individuals with DD, and 59 TD individuals.

Pupil data were exported from Tobii Studio and manipulated in SPSS version 24.0 (IBM Corporation, Armonk, NY, USA). For each participant, their pupil data were averaged across both eyes and then filtered to remove any outlier values related to blinks, loss of tracking data, large changes in head movement or if the participant did not look at the preceding scramble face image for three or more consecutive 250 ms intervals. Mean pupil diameter was calculated for interval durations of 250 ms across the scramble (1-s) and face presentation (5-s) for a total of 24 intervals. Consistent with Farzin et al. (2009, 2011), face specific pupil reactivity was calculated by subtracting the mean pupil size during the preceding scrambled face from the mean pupil size during each interval (*n* = 20) of the face presentation, and then “standardized” by dividing by the mean pupil size during the scrambled faces. Further, pupil reactivity was averaged across trials of each face emotion for test-retest reliability analyses within the subset of ASD participants.

#### Statistical Tests

Data were examined for outliers, nonnormality, and homoscedasticity. Since age was significantly different between groups with a wide age range within each group, age was included in all models as a covariate to account for these differences. Preliminary and the first set of analyses were completed in SAS® 9.4 (SAS Institute Inc., Cary, NC, USA). A mixed model analysis of covariance (ANCOVA) with random subject effects using AOI region, emotion, and group as the independent variables and proportion of looking as the dependent variable was conducted. Since fixation count was not normally distributed, a Poisson regression model, accounting for over-dispersion, using AOI region, emotion, and the group as the independent variables and fixation count as the dependent variable was conducted. Further, repeated measures ANCOVA with interval, emotion, and the group as the independent variables and pupil reactivity as the dependent variable was conducted. Within each model, significant main effects and interactions were followed up with least-square means to acquire adjusted mean differences. False Discovery Rate (Benjamini and Hochberg, 1995) was utilized to control for family-wise error in the *post hoc* analyses. In addition, adjustments were made for denominator degrees of freedom for all models (Kenward and Roger, 1997).

For the second set of analyses, R version 3.5.1 (R Foundation for Statistical Computing, Vienna, Austria) was utilized. To assess the test-retest reliability of the emotional faces paradigm with a subset of the ASD sample, we computed intraclass correlation coefficients (ICCs) between the two testing sessions using a two-way random-effects model with absolute agreement (ICC 2, 1; Shrout and Fleiss, 1979). The random-effects model is ideal because it allows for systematic differences between the two testing sessions. Further, ICC’s are better able to detect systematic differences between testing sessions in comparison to correlation coefficients (Weir, 2005). If participants performed similarly across the two testing sessions, their ICC will be closer to 1. Analyses focus on the ICCs for each AOI within fixation counts and proportion of looking. Pupil reactivity was averaged across intervals with ICCs reported on the different emotional faces that were presented. Definitive guidelines for interpreting ICC values have not been well justified; however there are a few documented guidelines like the tiered approach suggested by Cicchetti (1994): <0.40 = poor, 0.40–0.59 = fair, 0.60–0.74 = good, and 0.75–1.00 = excellent. Skinner et al. (2018) caution against using eye-tracking measures with reliability coefficients less than 0.60.

## Results

### Preliminary Analyses

#### Cognitive Abilities

We examined the relationship of cognitive ability on total data contribution to the eye-tracking task given the large amount of variability within the present study’s sample. Utilizing a median split for Full-Scale IQ to separate the entire sample, an independent measures *t*-test revealed a significant difference between groups in the proportion of attention to the eye-tracking task (*t*_(114)_ = −2.71, *p* = 0.008). Specifically, participants who had an IQ of >95 (*M* = 76.82, *SD* = 12.26) attended to the eye-tracking task more in comparison to those with an IQ ≤95 (*M* = 69.88, *SD* = 15.17). When looking within groups, these differences were minimized. In the TD sample, no significant differences were found in their proportion of attention to the task utilizing a median split of IQ (*t*_(57)_ = 1.74, *p* = 0.088). The TD participants who had an IQ of >103 (*M* = 79.26, *SD* = 10.13) attended to the task similar to those with an IQ ≤103 (*M* = 73.81, *SD* = 13.67). Within the DD group, there were no significant differences found utilizing a median split of IQ on proportion of attention to the task (*t*_(25)_ = 0.73, *p* = 0.472). The DD participants with an IQ of >76 (*M* = 72.99, *SD* = 14.71) attended to the task similar to those with an IQ ≤76 (*M* = 69.09, *SD* = 13.01). Within the ASD group, there were no significant differences found utilizing a median split of IQ on proportion of attention to the task (*t*_(28)_ = 1.42, *p* = 0.166). The ASD participants with an IQ of >86 (*M* = 73.79, *SD* = 17.68) attended the task similar to those with an IQ ≤86 (*M* = 65.15, *SD* = 15.52).

#### Age

We examined the relationship of age on total data contribution to the eye-tracking task given the large amount of variability within the present study’s sample. Utilizing a median split for age to separate the entire sample, an independent measures *t*-test revealed a significant difference between groups in the proportion of attention to the eye-tracking task (*t*_(121)_ = −2.24, *p* = 0.027). Specifically, participants >12.32 years (*M* = 75.54, *SD* = 15.07) attended to the eye-tracking task more in comparison to ≤12.32 years old (*M* = 69.69, *SD* = 13.93). When looking within groups, these differences were minimized for the ASD and DD groups. In the TD sample, significant differences were found in their proportion of attention to the task utilizing a median split of age (*t*_(57)_ = −2.59, *p* = 0.012). The TD participants >11.12 years (*M* = 80.64, *SD* = 9.08) attended to the task more than those ≤11.12 years old (*M* = 72.74, *SD* = 13.64). Within the DD group, there were no significant differences found utilizing a median split of age on the proportion of attention to the task (*t*_(26)_ = −1.41, *p* = 0.171). The DD participants >9.94 years (*M* = 74.65, *SD* = 12.87) attended to the task similarly to ≤9.94 years old (*M* = 67.59, *SD* = 13.64). Within the ASD group, there were no significant differences found utilizing a median split of age on the proportion of attention to the task (*t*_(34)_ = −0.67, *p* = 0.506). The ASD participants >16.41 years (*M* = 69.36, *SD* = 19.31) attended to the task similarly to ≤16.4 years old (*M* = 65.36, *SD* = 16.19).

### Proportion of Looking

**Figure 2:**
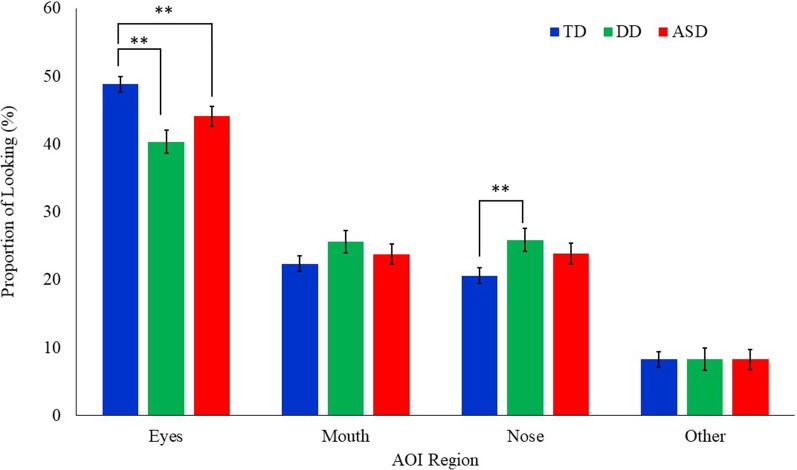
Mean proportion of looking to each AOI region by group for all emotional faces. Proportion of looking is reported as percentages and error bars represent SEM. TD, typically developing; DD, developmentally delayed; ASD, autism spectrum disorder; **p < 0.05.

**Figure 3:**
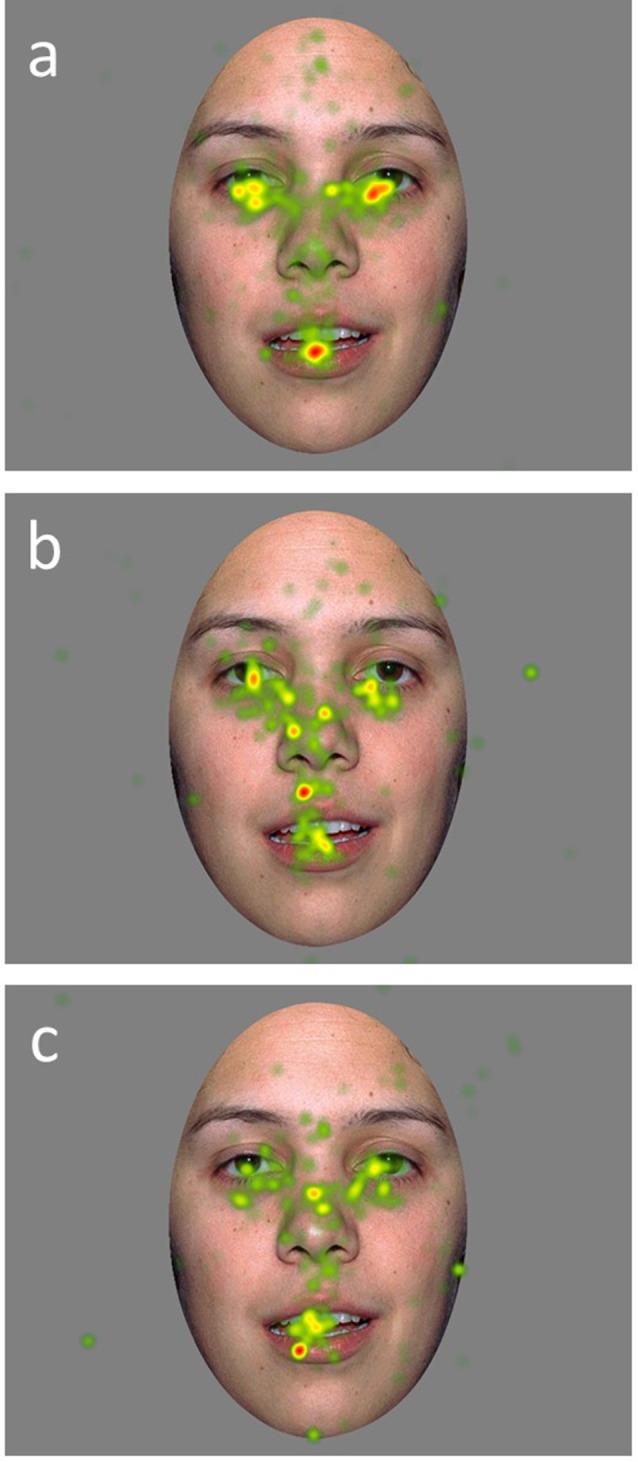
Heat maps of average duration fixation on a calm face image for a subset of TD **(A)**, DD **(B)**, and ASD **(C)**, participants.

A mixed model ANCOVA with AOI region, emotion, and the group as independent variables, age as a covariate, and proportion of looking as the dependent variable was conducted (Table 2). Results revealed a main effect of AOI region (*F*_(3,1439)_ = 307.85, *p* = 0.000). This effect was qualified by a significant interaction between AOI region and group (*F*_(6,1439)_ = 4.70, *p* = 0.001; Figure 2). Least squares mean differences revealed the TD participants (*M* = 48.82 *SE* = 1.17) spent significantly more time looking at the eyes in comparison to the DD participants (*t*_(1439)_ = 4.13, *p* = 0.000; *M* = 40.34, *SE* = 1.69) and the ASD participants (*t*_(1439)_ = 2.50, *p* = 0.013; *M* = 44.08, *SE* = 1.50). Further, the TD participants (*M* = 20.60, *SE* = 1.17) spent significantly less time looking at the nose in comparison to the DD participants (*t*_(1439)_ = −2.56, *p* = 0.011; *M* = 25.60, *SE* = 1.69) but similarly to the ASD participants (*t*_(1439)_ = −1.71, *p* = 0.087, *M* = 23.84, *SE* = 1.50). No other significant main effects or interactions emerged. See Figure 3 for a heat map of the average duration of looking for a subgroup of participants within each clinical group for one of the neutral faces.

**Table 2:** Results of mixed-model ANCOVA within-subjects effects for the proportion of looking.

Variable	*F*	*p*
Age	0.01	0.941
Group	0.00	0.999
Emotion	0.00	1.000
AOI	307.85	0.000*
Group × Emotion	0.00	1.000
Group × AOI	4.70	0.000*
Emotion × AOI	1.04	0.400
Group × Emotion × AOI	0.49	0.923

Note. AOI, area of interest. *p < 0.05.

### Fixation Count

**Figure 4:**
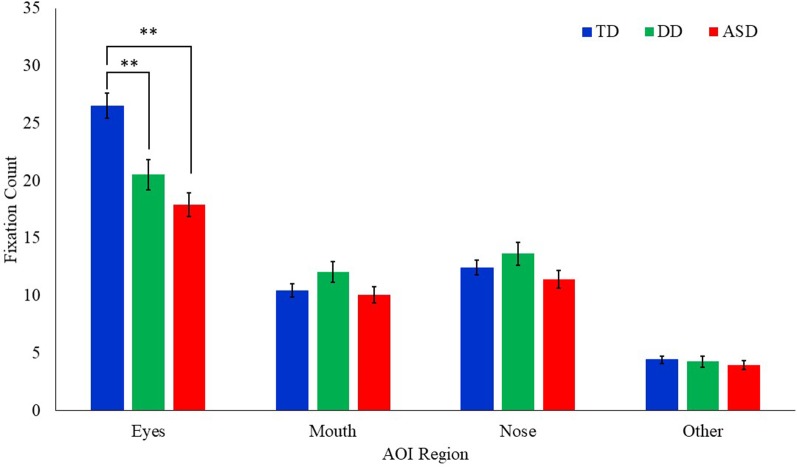
Mean fixation count to each AOI region by group for all emotional faces. Error bars represent SEM. TD, typical developing; DD, developmentally delayed; ASD, autism spectrum disorder; **p < 0.05.

A Poisson regression model with AOI region, emotion, and the group as the independent variables, age as a covariate, and fixation count as the dependent variable was conducted (Table 3). Results revealed a significant main effect of AOI region (*F*_(3,1317)_ = 300.61, *p* = 0.000) and group (*F*_(2,151.7)_ = 3.35, *p* = 0.038). These effects were qualified by a significant interaction between group and AOI region (*F*_(6,1317)_ = 5.64, *p* = 0.000; Figure 4). Least square mean differences revealed the TD participants (*M* = 26.52, *SE* = 1.10) exhibited significantly more fixations on the eyes in comparison to the DD (*t*_(235.1)_ = 3.37, *p* = 0.001; *M* = 20.53, *SE* = 1.32) and the ASD (*t*_(243.2)_ = 5.48, *p* = 0.000; (*M* = 17.91, *SE* = 1.05) participants. No other significant main effects or interactions emerged.

**Table 3:** Results of Poisson regression for fixation count.

Variable	*F*	*p*
Age	14.33	0.001*
Group	3.35	0.037*
Emotion	1.83	0.162
AOI	300.61	0.000*
Group × Emotion	0.35	0.841
Group × AOI	5.64	0.000*
Emotion × AOI	1.36	0.228
Group × Emotion × AOI	0.65	0.803

Note. AOI, area of interest. *p < 0.05.

### Pupil Reactivity

**Figure 5:**
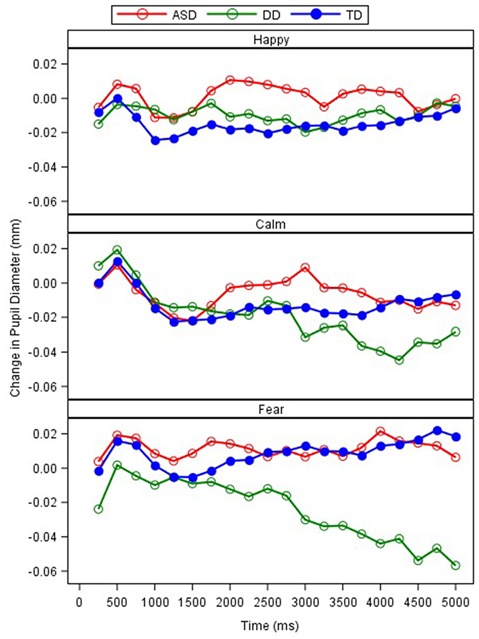
Relative change in pupil diameter (mm) between scrambled face to calm, happy, and fearful faces across 250-ms intervals, by the group.

A repeated measures ANCOVA with interval (*n =* 20), emotion, and group as independent variables, age as a covariate, and pupil reactivity as the dependent variable was conducted (Table 4). Results revealed a significant main effect of emotion (*F*_(2,11398)_ = 15.36, *p* = 0.000) and interval (*F*_(19,11305)_ = 2.28, *p* = 0.001). A marginally significant effect for group also emerged (*F*_(2,81.24)_ = 2.67, *p* = 0.075). These effects were qualified by a significant interaction between group and emotion (*F*_(4,11398)_ = 14.81, *p* = 0.000; Figure 5). Least square mean differences revealed the DD participants exhibited a significant reduction in pupil diameter during fearful faces in comparison to TD (*t*_(107.8)_ = −3.88, *p* = 0.000) and ASD *t*_(107.8)_ = 3.89, *p* = 0.000) participants. Additionally, the ASD participants exhibited a significant increase in pupil diameter during happy faces in comparison to TD participants (*t*_(104.3)_ = 2.35, *p* = 0.021). Additionally, a marginally significant interaction emerged between diagnosis and interval (*F*_(38,11305)_ = 1.35, *p* = 0.073). Least square mean differences revealed the interaction was being driven by the DD group on average exhibiting a significant reduction in pupil diameter across the last five intervals in comparison to the TD group (*p*s = 0.009–0.042). In contrast, the ASD group on average exhibited a significant increase in pupil reactivity across the last nine intervals in comparison to the DD group (*p*s = 0.011–0.047).

**Table 4:** Results of repeated-measures ANCOVA within-subjects effects for pupillary reactivity.

Variable	*F*	*p*
Age	2.21	0.141
Group	2.67	0.075
Emotion	15.36	0.000*
Interval	2.28	0.001*
Group × Emotion	14.81	0.000*
Group × Interval	1.35	0.073
Emotion × Interval	0.65	0.953
Group × Emotion × Interval	0.41	1.000

Note. AOI, area of interest. *p < 0.05.

### Test-Retest Reliability in ASD

Test-retest reliability was assessed using ICCs between the two testing sessions for the ASD participants for fixation counts, the proportion of looking, and pupil reactivity (Table 5). A good degree of reliability was found for the majority of the AOIs based on the proportion of looking (ICCs = 0.62–0.68). The proportion of looking time at the nose fell in the fair range (ICC = 0.50). Within the AOIs for fixation counts, a fair degree of reliability was found for the nose, mouth, and scrambled regions (ICCs = 0.40–0.56) with the eye region exhibiting a poor degree of reliability (ICC = 0.39). Lastly, changes in pupil reactivity demonstrated poor to fair reliability across the different emotional faces that were presented. Pupil reactivity to the fear faces demonstrated the largest reliability coefficient (ICC = 0.54). Reliability coefficients for change in pupil reactivity within calm faces were not reported due to the variability within the testing sessions being greater than across sessions, resulting in a negative ICC value.

**Table 5:** Test-retest reliability as measured by ICC calculations of eye-tracking measures between test sessions in ASD.

Variable	ICC (2, 1)	95% CI
Proportion of Looking		
Eyes	0.66	0.29–0.85
Nose	0.50	0.09–0.77
Mouth	0.68	0.35–0.86
Scramble	0.62	0.25–0.83
Fixation Count		
Eyes	0.39	−0.09–0.71
Nose	0.56	0.15–0.80
Mouth	0.45	0.02–0.74
Scramble	0.40	−0.07–0.72
Pupil Reactivity		
Calm	-	-
Happy	0.31	−0.18–0.71
Fear	0.54	0.00–0.85

*Note. ICC (2,1), Interclass Correlation Coefficient using a two-way random-effects model with absolute agreement; ASD, autism spectrum disorder; CI, confidence interval; ICC values for calm were not reported due to significant variability resulting in negative values*.

## Discussion

Social communication deficits are a hallmark characteristic of the ASD phenotype (American Psychiatric Association, 2013). More specifically, social attention deficits (e.g., reduced attention to social stimuli as a whole or atypical allocation of attention to social stimuli) within ASD have been hypothesized to have cascading effects on emotion recognition (Pelphrey et al., 2002; Wagner et al., 2013). With social attention deficits being a primary early biomarker for diagnostic outcomes in infancy (Jones and Klin, 2013; Elsabbagh et al., 2014; Jones et al., 2016), this is an ideal skill area for targeted assessment and treatment to potentially increase quality of life in individuals with ASD. Through precise, noninvasive measures like eye-tracking, the literature has shown promise in accurately identifying deficits in social attention and emotion recognition, but the sensitivity of the mechanism for treatment outcomes remains uncertain (Bradshaw et al., 2015). The present study aimed to assess the utility of an eye-tracking paradigm to discriminate between clinical groups in social attention and emotion recognition through face scanning and pupillometry. The present study also assessed the reliability of this paradigm within the ASD sample to further our understanding of the utility of eye-tracking for future clinical trials.

As expected, our analyses align with previous research (Klin et al., 2002; Pelphrey et al., 2002; Corden et al., 2008; Riby and Hancock, 2008; Rice et al., 2012; Hanley et al., 2013; Auyeung et al., 2015) suggesting atypical attention allocation to social stimuli in ASD; however, these differences were not distinguishable by varying emotions based on face scanning patterns and were confined to one area of the face. Specifically, the ASD group spent less time and fixated less on the eye region across all emotions in comparison to the TD control group. Moreover, differences in attention to the eye region between the ASD and DD groups was unclear, leaving the question of whether these atypical social attention profiles are ASD specific or related to cognitive functioning. Previous research examining attention to faces in clinical populations with known cognitive deficits (e.g., fragile × syndrome, Williams syndrome, Angelman syndrome) have also found reduced attention to the eye region of faces (Farzin et al., 2009, 2011; Riby and Hancock, 2009; Hong et al., 2017); however, the use of idiopathic mental-age matched comparison groups in the current literature is scarce, adding to the uncertainty of these deficits being syndrome specific or related to cognitive functioning. Further, the proportion of looking time spent on the nose in the DD group emerged as a region of interest. In comparison to the TD control group, the DD group spent more time looking at the nose region. The social attention profile of reduced looking to eyes and increased looking to nose may be notable for those with low IQ. Since our ASD sample had a wide range of IQ scores, it’s unclear in the current study if the visibly, but not statistically significant, elevated attention to the nose region in the ASD group is being driven by those with lower cognitive abilities. Although the preliminary analyses did not suggest differences in overall attention to the eye-tracking task based on IQ in the ASD group, it may be important in the future to examine if different social attention patterns emerge dependent on cognitive functioning. Notably, despite finding these subtle differences in social attention allocation for specific facial regions, the ASD group still exhibited a relatively similar social attention profile overall in comparison to the control groups (e.g., most time spent looking at eyes, less at nose, mouth, and other).

As anticipated, pupil reactivity was able to detect differences within the clinical groups based on the emotional faces that were presented. Unlike the findings presented by Sepeta et al. (2012), we found increased pupil reactivity in the ASD group when examining happy faces in comparison to the TD group. The TD group and DD group exhibited similar pupil reactivity profiles to the happy faces suggesting an ASD phenotype-specific response. Nonetheless, these findings conflict with the idea of abnormal social reward processing in ASD as measured through pupillometry. Based on the social motivation hypothesis, it is suggested that individuals with ASD will not attend to social stimuli because they do not form representations of the reward value of social stimuli (Dawson et al., 2005; Chevallier et al., 2012). Therefore, individuals with ASD will not seek out social stimuli because eye contact and faces are not intrinsically rewarding and may not be activating those cognitive reward systems appropriately. With the lack of facial scanning differences across emotions and increased pupil reactivity to the happy faces in the ASD group, additional work is needed to explore these findings and how they relate to emotion and social reward processing.

Unexpectedly, the ASD group exhibited similar pupil reactivity profiles to the calm and fear faces in comparison to the TD group. Unlike the findings presented by Nuske et al. (2014a), both the TD and ASD groups exhibited a slight increase in pupil diameter while viewing fearful faces. These findings may be explained by the lack of significant change in pupil diameter exhibited by our TD group that was found by Nuske et al. (2014a) as both the ASD and TD group in the present study partially resemble their ASD findings. Further, the paradigm that was utilized was slightly different as we did not strategically show neutral faces right before the fearful faces. However, our findings partially replicate previous work (Sepeta et al., 2012; Wagner et al., 2013) suggesting no group differences in pupil diameter in response to emotional faces. It is quite possible that emotion processing is better understood utilizing multiple mechanisms of autonomic activity. Bradley et al. (2008) utilized measures of pupillometry, heart rate, and skin conductance in a group of TD individuals who viewed emotional faces. Through these mechanisms, they were able to strongly support that pupil reactivity in response to emotionally-salient faces was moderated by the sympathetic system. Although many groups have been able to identify ASD specific emotion processing through pupillometry alone, a multimethod physiological approach may be warranted to delineate the mixed findings.

Aside from the group differences in social attention and emotion processing, the present study also examined the test-retest reliability of the emotional faces paradigm that was utilized in the ASD group. Test-retest reliability considers the variability between individuals’ repeated measurements relative to the overall group variance (de Vet et al., 2006). Farzin et al. (2009, 2011) reported high reliability of an extended version of the paradigm in a small sample of FXS participants. Our reliability estimates were less promising based on the mechanism assessed while aligning with the known variability of the ASD phenotype and mixed literature supporting eye tracking as a reliable biomarker for treatment change. Specifically, our results found the highest reliability estimates through the proportion of looking time at the mouth or the eyes. The number of fixations across AOIs and pupil reactivity to the different emotions resulted in poor to fair reliability coefficients. The low ICCs found for some of the eye-tracking variables suggest they may not be appropriate for discriminative testing when comparing across groups and caution should be placed when interpreting the results of those specific AOIs. Of note, previous literature suggests higher reliability coefficients are more likely to occur from longer trial duration (Skinner et al., 2018). This may explain why our reliability estimates were not as strong as those reported by Farzin et al. (2011) because they administered more face trials than administered in the present study. The reliability estimates reported may have been boosted if the paradigm lasted longer and presented more faces; however, when working with individuals with neurodevelopmental disabilities, long eye-tracking tasks can be challenging to complete while still obtaining adequate and useable data.

In order to consider the validity of eye-tracking as a biomarker in ASD, we must also consider how the typical sample in the present study aligns with the current literature on social attention development. There is a robust amount of literature indicating that when TD individuals are presented with photos or videos of people, they are drawn to look at people rather than objects, with a particular focus on the eye region (e.g., for review, see Frischen et al., 2007; Birmingham and Kingstone, 2009). The facial scanning patterns of the present study’s TD sample aligns with the current literature on social attention. Specifically, the TD sample predominantly attended to the eye region of faces as demonstrated through the overall proportion of looking time and fixation counts. As for pupillometry responses, there have been consistent findings in the TD literature indicating emotional stimuli, in comparison to neutral stimuli, produces greater pupillary responses (Henderson et al., 2014; Cohen et al., 2015). Although the focus of the present study was on group differences in pupillometry responses to emotional stimuli, resulting in the TD groups not being significantly different, our TD sample visually appears to demonstrate a slight increase in pupil size across the presentation of the different emotional faces. Specifically, the TD sample had a stronger reaction to fear faces compared to happy faces in comparison to calm faces. Therefore, the present study’s findings within the TD sample align with previous literature on typical social attention profiles suggesting these findings build on the validity of the eye-tracking paradigm used, the interpretation of the findings within ASD and DD, and the utility of eye-tracking as a biomarker.

Overall, these findings continue to build on the promise of eye-tracking as a feasible and reliable biomarker for identifying social attention and emotion recognition deficits in ASD. This may be less apparent for detecting emotion recognition explicitly through facial scanning within ASD. However, the combined mechanisms of pupillometry and facial scanning provided more precision in the present study for understanding the social attention and emotion recognition profiles in a chronologically and cognitively diverse ASD group. Furthermore, the present study attempted to rule out the effects of IQ with the addition of an idiopathic mental-age matched control group. Unfortunately, an ASD specific social attention profile was not as clearly delineated given the lack of group differences between the mental-age matched control group and the ASD group. Notably, eight of our participants were not able to complete cognitive testing; however, six of the eight were able to complete the eye-tracking task with at least 35% valid looking data, which allowed for our sample to be cognitively diverse. Cognitive functioning did not present as a factor impairing overall attention during the task within the clinical groups. This suggests that regardless of cognitive functioning, these clinical groups were able to successfully complete the task and that there are potentially salient social attention profiles specific to cognitive abilities to be further explored rather than concerns with general attention in these clinical populations.

The utility of the emotional faces eye-tracking paradigm assessed in the present study should continue to be evaluated given the wide range of test-retest reliability coefficients reported, in addition to other eye-tracking paradigms that are widely used in the literature that have been shown to consistently distinguish between clinical groups. More recently, a clinical trial utilizing an extended version of the emotional faces paradigm suggested the paradigm was sensitive enough to detect increases in overall looking time, fixations, and pupil reactivity in adolescents and adults with FXS (Hessl et al., 2019). Since many individuals with FXS also receive a co-occurring diagnosis of ASD (Klusek et al., 2014; Talisa et al., 2014; Thurman et al., 2014), the emotional faces paradigm may be sensitive enough to detect a change in treatment trials targeting social and emotional impairments in idiopathic ASD. The specific mechanisms within the eye-tracking paradigm (e.g., proportion of looking vs. fixation counts) may be more or less viable for assessing treatment-specific outcomes that are lacking in the current literature.

Despite the many strengths of the present study, there are also limitations to consider when interpreting the findings. Specifically, age consistently presented as a significant variable in the analyses. Since our age range was quite wide (3–25 years), as well as IQ within the ASD sample, it may be important for future researchers to look at subgroup responses based on age and cognitive abilities as the paradigm utilized may be a better biomarker and outcome measure for certain subgroups within the different clinical groups. For example, differences in maturation in social attention within the clinical groups could be a driving factor of the group differences that emerged. Additionally, the reliability analyses were reported with a small subgroup of the ASD sample who had test-retest data available. Future work should continue to explore test-retest reliability in ASD utilizing a larger sample with a goal of identifying the ideal necessary length or amount of trials needed in an eye-tracking paradigm in this population. It would also be important to compare the test-retest reliability estimates across clinical groups as the reliability estimates reported in the present study could be specific to the variability in the ASD phenotype or the eye-tracking measure utilized. Future work should examine test-retest reliability within multiple clinical samples to further clarify these findings. Also, the present study utilized static photographs of faces to examine social attention and emotion recognition. The use of dynamic social stimuli that resemble real-life social situations could extend these findings. With the lack of differences between the emotions presented in the paradigm, as mentioned above, expanding the paradigm to included more faces may have provided additional power to find different social attention patterns for each of the emotions. Further, the present studies paradigm did not map onto the racial and ethnic diversity of the study’s sample. Lastly, incorporating additional physiological (e.g., heart rate, skin conductance) or electrophysiological measures to assess social attention and emotion recognition from a biobehavioral perspective may provide a more sensitive model for assessing deficits and change across treatment while delineating some of the variability in the literature.

## Data Availability Statement

Written informed consent was obtained from the [individual(s) AND/OR minor(s)’ legal guardian/next of kin] for the publication of any potentially identifiable images or data included in this article.

## Ethics Statement

The studies involving humans were approved by the Cincinnati Children’s Hospital Medical Center Institutional Review Board. Written informed consent was obtained from the patients/participants’ legal guardian/next of kin. Standard Operating Procedures of the IRB were followed to evaluate if assent from participants was possible. This was carried out on a case by case basis, and was obtained where feasible.

## Author Contributions

CE, RS, KD, and EP contributed to study conceptualization and data collection. CE, RS, MH, and DR contributed to the manuscript preparation and revisions. DR led the writing of the manuscript. MH contributed to data extraction and preparation. DR and PH analyzed and interpreted the data.

## Conflict of Interest

RS receives funding from Fulcrum Therapeutics. CE has received current or past funding from Confluence Pharmaceuticals, Novartis, F. Hoffmann-La Roche Limited, Seaside Therapeutics, Riovant Sciences, Inc., Fulcrum Therapeutics, Neuren Pharmaceuticals Limited, Alcobra Pharmaceuticals, Neurotrope, Zynerba Pharmaceuticals, Inc., Lenire Bioscience, and Ovid Therapeutics Inc. to consult on trial design or development strategies and/or conduct clinical trials in FXS or other neurodevelopmental disorders. CE is additionally the inventor or co-inventor on several patents held by Cincinnati Children’s Hospital Medical Center or Indiana University School of Medicine describing methods of treatment in FXS or other neurodevelopmental disorders. EP has received research support from the National Institutes of Health (NIMH), American Academy of Child and Adolescent Psychiatry, and Cincinnati Children’s Hospital Research Foundation. He is a clinical trial site investigator for the Marcus Autism Center (clinical trial, Autism). He receives compensation for consulting for Proctor and Gamble and Eccrine Systems, LLC. He receives book royalties from Springer. There are no conflicts of interest with the current manuscript. KD has received research support from the National Institute of Neurological Disorders and Stroke (NINDS), American Academy of Child and Adolescent Psychiatry, and Cincinnati Children’s Hospital Medical Center. She is a clinical trial site investigator for F. Hoffman-La Roche Limited, and Ovid Therapeutics. The remaining authors declare that the research was conducted in the absence of any commercial or financial relationships that could be construed as a potential conflict of interest.
